# Addition of CFCl_3_ to Aromatic Aldehydes via *in Situ* Grignard Reaction

**DOI:** 10.3390/molecules200815098

**Published:** 2015-08-18

**Authors:** Balaka Barkakaty, Bandana Talukdar, Bradley S. Lokitz

**Affiliations:** 1 Center for Nanophase Materials Sciences, Oak Ridge National Laboratory, One Bethel Valley Road, Oak Ridge, TN 37831, USA; E-Mail: lokitzbs@ornl.gov; 2 Independent Researcher, Juripar, Panjabari, Guwahati 781037, Assam, India; E-Mail: drbandana.t@gmail.com

**Keywords:** CFCl_3_, magnesium, Grignard reaction, *in-situ* process, aromatic aldehydes

## Abstract

Synthetic modification of trichlorofluoromethane (CFCl_3_) to non-volatile and useful fluorinated precursors is a cost-effective and an environmentally benign strategy for the safe consumption/destruction of the ozone depleting potential of the reagent. In this report, we present a novel method for *in situ* Grignard reaction using magnesium powder and CFCl_3_ for synthesis of dichlorofluoromethyl aromatic alcohols.

## 1. Introduction

Chlorofluorocarbons (CFCs), commonly known as “Freons” are organic compounds mainly composed of carbon, chlorine and fluorine, and are structural derivatives of methane, ethane and propane. These compounds are non-toxic, non-flammable and do not react easily with other compounds. Due to their inert properties, they gained huge industrial interest starting in the 1930s as safe, non-toxic, and non-flammable alternatives to hazardous chemicals such as ammonia, methyl chloride, and sulfur dioxide for use in refrigerants or spray can propellants [1]. During the late 1950s and early 1960s, the CFCs were used as a cost-effective solution to provide air conditioning to automobiles, homes, and office buildings. Later, the growth of the CFC market saw an exponential rise, and the annual sales of CFCs reached about a billion dollars (US), and more than one million metric tons of CFCs were produced [2]. In 1974, Professor Sherwood Rowland and Dr. Mario Molina from the University of California reported that chlorine released from CFCs was responsible for the rapid destruction of the ozone layer by creating ozone holes in the stratosphere. It was found that one chlorine atom released from CFCs destroyed 100,000 molecules of ozone [2]. In 1987, this discovery eventually led to a global environmental treaty, The Montreal Protocol, signed by 27 nations to reduce substances that deplete the ozone layer [3]. Later, in 1990, an amendment was approved in London for complete elimination of CFC production by 2000 [2]. However, the sudden elimination of CFCs from the market created a huge reserve of CFC waste worldwide. This necessitated a demand for safe storage or utilization of the CFCs in a recyclable fashion or destruction through expensive thermal plasma treatments [4]. This scenario led to the development of a new research field, *i.e.*, cost-effective and synthetic utilization of CFCs for the synthesis of novel and non-hazardous fluorinated compounds [5,6,7,8,9,10,11,12,13]. Out of the many CFCs, CFCl_3_ or trichlorofluoromethane (CFC-11) has the maximum ozone depletion potential [14]. In 2006 and 2007, we reported novel techniques for the reductive addition of trichlorofluoromethane (CFCl_3_) to aromatic aldehydes by utilizing an activated aluminum/tin chloride (II) system under ultrasonic irradiation [15,16]. To our knowledge, these were the first reports to provide simple and feasible synthetic methods to generate a class of α-fluorinated aryl ketones. We reported that using an aluminum/tin chloride (II) system resulted in low to medium yields unless ultrasonic irradiation was used [15]. Other metal catalysts also failed to increase the yield even under elevated temperature conditions [17]. Our method employed 1.25 equivalents of tin chloride (II) and 3.0 equivalents of aluminum under ultrasonic irradiation to drive the reaction [15]. However, aluminum [18] and tin chloride (II) [19] are considered to be toxic and hazardous. Compared to aluminum and tin chloride (II), magnesium has much reduced toxicological effects [20,21]. In an attempt to develop a greener alternative to our previous aluminum/tin chloride (II) system, we herein report a novel, milder and facile method to produce dichlorofluoromethyl aromatic alcohols through the addition of CFCl_3_ to aromatic aldehydes using only magnesium powder.

## 2. Results and Discussion

Prior to our previous report [15], the addition of CFCl_3_ to carbonyl groups was mainly constrained to highly activated carbonyl compounds such as perfluoro carboxylic acid esters and aromatic carbonyl entities attached to fluoro or trifluoromethyl moieties [9,17]. Even after activation with trifluoromethyl or fluoro aromatic substituents, the isolated product yields were only modest (58%–79%) and low (39%) for un-activated substrates such as benzaldehdye [9,17]. Hu *et al.* succeeded in obtaining dichlorofluoromethyl alcohols by reaction of CFCl_3_ with less electrophilic ketones using magnesium/lithium chloride as catalysts [22]. However, these reactions were extremely sensitive to temperature, requiring a stringent temperature range between −20 °C to −15 °C to be maintained, and isolated product yields were only of 45%–62%. It was found that even at 0 °C, the reaction yielded various side products with pinacol as the major component. We attributed these results to the formation of an *in situ* highly reactive organometallic complex by reaction of magnesium with CFCl_3_ in the presence of strong lewis acid such as LiCl under very low temperature conditions. The high reactivity of the *in situ* complex provided the driving force for reactions with even less electrophilic ketones, but the complex was unstable and decomposed at temperatures even lower than 0 °C. In support of our initial assumptions about LiCl activation, recent studies have verified that addition of LiCl to *i*PrMgCl generates a more active reagent *i*PrMgCl∙LiCl which is superior in reactivity to Grignard reagents [23,24]. Furthermore, in support of the hypothesis for *in situ* reactions, previous studies have demonstrated that Grignard reagents produced from α-haloalkyl compounds are highly reactive and are usually generated from reactions under *in situ* conditions [25]. Therefore, considering the probability of *in situ* formation of a relatively more stable dichlorofluoro-magnesium Grignard reagent under milder conditions, we attempted the reaction of CFCl_3_ with aromatic aldehydes in the presence of only magnesium powder in dimethylformamide (DMF) at room temperature.

**Scheme 1:**
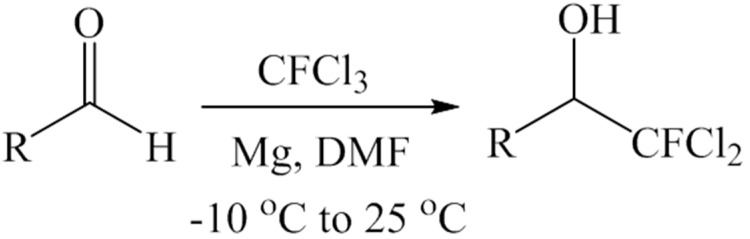
Synthesis of dichlorofluoromethylaryl alcohols from aromatic aldehydes.

In this system, we have used magnesium powder (1 equivalent) and CFCl_3_ (3.0 equivalent) in DMF to carry out the desired reaction (Scheme 1, Table 1). An excess of CFCl_3_ was needed because of its high volatility at room temperature (boiling point of CFCl_3_ = 23.77 °C). It must be noted that the reaction is extremely sensitive to moisture, and the yield decreases drastically in the presence of trace amounts of moisture in the solvent, reagents or apparatus used for the reaction.

**Table 1:** Magnesium activated addition of CFCl_3_ to aromatic aldehydes ^a^.

Entry	Substrate 1	Time (h)	Product 2	Yield (%)
1	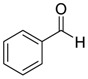 **1a**	4	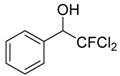 **2a**	55 ^b^
2	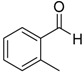 **1b**	4	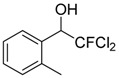 **2b**	50 ^b^
3	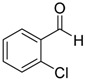 **1c**	3	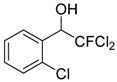 **2c**	20 ^b^
4	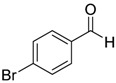 **1d**	3	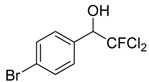 **2d**	14 ^b^
5	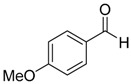 **1e**	4	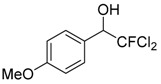 **2e**	47 ^b^
6	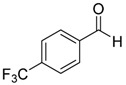 **1f**	4	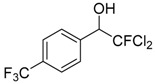 **2f**	65 ^b^
7	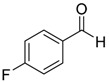 **1g**	4	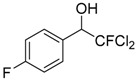 **2g**	60 ^b^
8	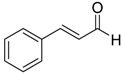 **1h**	4	unknown mixture ^c^	
9	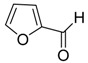 **1i**	4	unknown mixture ^c^	

^a^ Carried out with aldehydes **1** (1.0 equiv.), Mg powder (1 equiv.), CFCl3 (3.0 equiv.) in DMF. ^b^ Isolated yields based on starting material (aldehydes **1**) consumed. ^1^H-NMR yields ^c^.

**Scheme 2:**
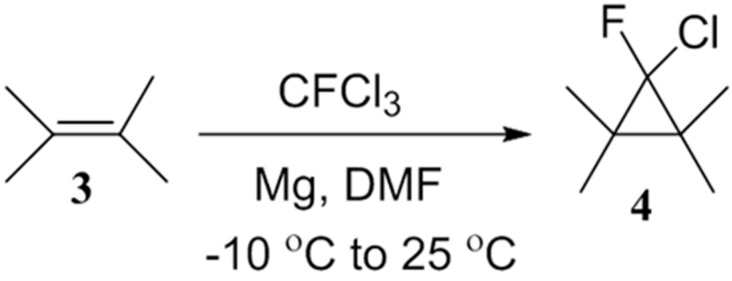
Addition of CFCl_3_ to tetramethylene (**3**) in presence of magnesium powder.

As shown in Table 1, the overall yields of the products under these conditions are lower than our previous report [15]. We attribute this to the equal probability of forming chlorofluorocarbene under these conditions as reaction of CFCl_3_ with tetramethylethylene (**3**) under the same conditions gave 1,1,2,2-tetramethylchlrofluorocyclopropane (**4**, 60% yield) as shown in Scheme 2. This finding is similar to the work as reported by Hu *et al.* [22] and also to the work reported by Lin *et al.* [26] where reaction of CFCl_3_ [22] or CCl_4_ [26] with magnesium has been reported to generate chlorofluorocarbene and dichlorocarbene respectively.

**Scheme 3:**
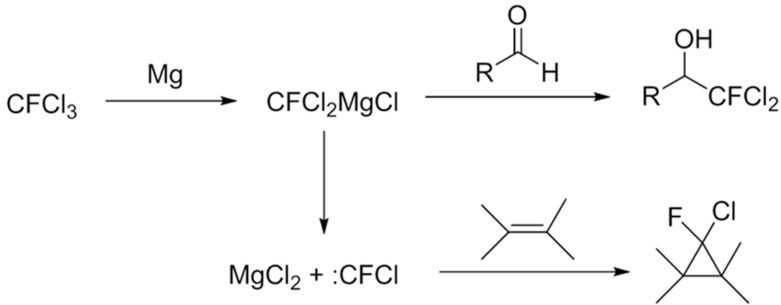
Proposed mechanism for reaction of CFCl_3_ with aromatic aldehydes or alkenes in presence of magnesium powder.

Formation of chlorofluorocarbene was also reported as the major reaction pathway in the reaction of CFCl_3_ with reduced titanium [27]. Burton *et al.* have investigated in detail the mechanism for reaction of cadmium or zinc metal with difluorodihalomethanes in DMF and have reported that the reaction proceeds through the formation of a difluorohalomethide anion intermediate followed by its decay to difluorocarbene [28].The difluorocarbene subsequently reacts with a fluoride anion provided by difluoromethylamine (a product of DMF and difluorocarbene) to form a trifluoromethyl carbanion of cadmium or zinc reagents [28]. Under our reaction conditions, the formation of chlorofluomethylamine could not be detected in order to identify an analogous stepwise mechanism for formation of dichlorofluoromethyl magnesium reagents. However, similar to the mechanism as proposed by Hu *et al.* [22], and Burton *et al.* [28]. we postulate that under our reaction conditions at −10 °C to 25 °C (~room temperature), a highly reactive dichlorofluoromethyl magnesium is formed which subsequently reacts with the carbonyl group on the aromatic aldehyde to give the dichlorofluomethyl aromatic alcohols. However, because the highly reactive dichlorofluomethyl carbenoid can also undergo a competing transformation reaction to form chlorofluorocarbene, the desired chlorofluoromethyl aromatic alcohols are therefore obtained in lower yields. A proposed reaction mechanism is shown in Scheme 3.

Carbenoid intermediates containing fluorine substituents are highly unstable, and methods for obtaining higher yields of products using these intermediates have been successful only in cases where the carbonyl groups are activated by electron withdrawing groups [9,17] or by employing unconventional activating methods such as ultrasonic irradiation [15]. Temperature also plays a very important role in these reactions [22,29]. Similar to temperature sensitive reactions of CFCl_3_ with carbonyl compounds as reported by Hu *et al.* [22] and as described earlier, Burton *et al.* also have reported that fluorinated carbenoids of lithium are highly reactive and are only stable at −116 °C and that yields of cyclopropane formed by the reaction of a fluorinated carbene to an alkene significantly drops from 49% to 35% when the temperature is increased to −78 °C [29]. Compared to previous reports [22,28,29], our method (Scheme 1, Table 1) utilizes milder temperatures (−10 °C to 25 °C) and yields a stable dichlorofluoromethyl carbenoid or dichlorofluoromethyl-magnesium Grignard reagent under *in situ* conditions. Lowering the reaction temperature did not increase the yield.

In contrast to our previous report [15], it is interesting to note that although the product yield increases when the phenyl ring is activated by electron withdrawing substituents such as -F or -CF_3_, the yields decrease when the phenyl ring is substituted with other electron withdrawing halogen moieties such as -Cl or -Br but not -F. On the contrary, for aromatic aldehydes with electron donating substituents such as 2-Me or 4-OMe, the yields of the corresponding products were higher than that with -Cl or -Br substituents. It was found that the products with -Cl and -Br substituents were also accompanied by mixtures of unknown side products as determined by ^1^H-NMR analyses. The lower yields and accompanying side reactions in the case of -Cl or -Br substituted aldehydes might be a result of Br/Mg or Cl/Mg exchange of the starting aldehyde or the generated intermediates with the highly reactive dichlorofluoromethyl magnesium reagent formed *in situ*. Reaction mixtures obtained from reactions of 1-cinnamaldehyde and 2-furaldehyde were very complex and could not be separated or identified.

## 3. Experimental Section

### 3.1. General

^1^H-NMR, ^13^C-NMR (internal standard: tetramethylsilane) and ^19^F-NMR (internal standard: C_6_F_6_) spectra were recorded on a JEOL JNM-AL300 spectrometer. Fourier transform infrared (FTIR) spectra were recorded on a Thermo Nicolet Avatar 360T2 infrared spectrophotometer. Elemental analyses were performed on a Perkin-Elmer 2400 series II CHNS/O analyzer. For thin layer chromatography (TLC), aluminum sheets, Merck silica gel coated 60 F254 plates were used, and the plates were visualized with UV light and phosphomolybdic acid (5% in EtOH). Merck silica gel 60 N (spherical, neutral) (40–50 µm) was used for the flash chromatography.

### 3.2. Materials

Trichlorofluoromethane (CFCl_3_) was obtained from Sigma-Aldrich and stored in the freezer over 4 Å molecular sieves. It is worth mentioning here that the boiling point of CFCl_3_ is 23.77 °C and, therefore, should be stored under cold conditions. Fresh magnesium powder was obtained from Sigma-Aldrich and was used without further purification. Fresh anhydrous DMF was obtained by distillation from calcium hydride and was stored over 4 Å molecular sieves.

### 3.3. Method: Activation of CFCl_3_ by Magnesium Powder and Its Addition to Aromatic Aldehydes-Typical Procedure

A two-necked flask equipped with a reflux condenser was flame dried and flushed with argon. Then, magnesium powder (230 mg, 9.4 mmol) followed by anhydrous DMF (25 mL) was added to the flask under argon atmosphere. Then, the flask was cooled to −10 °C using ice/acetone mixture. CFCl_3_ was then added to the cooled reaction mixture via a cannula and the combined reaction mixture was stirred at −10 °C for 10 min under an argon atmosphere. During this initial stirring, heat was evolved and magnesium powder in the reaction mixture was slowly consumed thereby indicating the formation of the Grignard complex. After 10 min, benzaldehyde (990 mg, 9.4 mmol, 0.96 mL) was added to the reaction flask via a syringe under an argon atmosphere at −10 °C. Then, the reaction mixture was stirred and allowed to come to room temperature for 4 h. The reaction was quenched with 5% aqueous HCl solution, and the mixture was extracted five times with ethyl acetate. The combined extracts were washed with an aqueous solution of NaHCO_3_ and brine followed by drying the organic layer over anhydrous MgSO_4_. The volatile organic solvent was then removed by rotary evaporation. The crude product was purified by column chromatography (Hexane/EtOAc = 3:1) to give 2,2-dichloro-2-fluoro-1-phenylethanol (1.10 g; 55% yield). Other compounds were also obtained by following similar procedures. As mentioned earlier, this reaction is extremely sensitive to moisture and should be performed under stringent dry conditions. We used freshly bought magnesium powder for our reactions. Old stock of magnesium powder might also work well for these reactions considering the high reactivity of α-haloalkyl Grignard reagents, or, alternatively, ultrasonic activation of magnesium powder in DMF before addition of CFCl_3_ might be a good option.

### 3.4. Characterization of the Products

All the products, **2a**–**e** were identified by comparison of spectroscopic data (IR, ^1^H-NMR, ^13^C-NMR and ^19^F-NMR) with the authentic samples as reported in [15,30,31]. The characteristic ^1^H-NMR values are given below for reference:

*2,2-Dichloro-2-fluoro-1-phenylethanol* (**2a**). ^1^H-NMR (CDCl_3_): δ = 7.53 (m, 2H), 7.41 (m, 3H), 5.16 (d, *J* = 8.4 Hz, 1H), 2.97 (br s, 1H, OH).
*2,2-Dichloro-2-fluoro-1-(2-methylphenyl)ethanol* (**2b**). ^1^H-NMR (CDCl_3_): δ = 7.66 (d, *J* = 6.9 Hz, 1H), 7.25 (m, 3H), 5.43 (d, *J* = 9.3 Hz, 1H), 2.60 (s, 3H), 3.05 (br s, 1H, OH).
*2,2-Dichloro-2-fluoro-1-(2-chlorophenyl)ethanol* (**2c**). ^1^H-NMR (CDCl_3_): δ = 7.75 (m, 1H), 7.36 (m, 3H), 5.77 (d, *J* = 9.3 Hz, 1H), 3.17 (br s, 1H, OH).
*2,2-Dichloro-2-fluoro-1-(4-bromophenyl)ethanol* (**2d**). ^1^H-NMR (CDCl_3_): δ = 7.53 (m, 2H), 7.40 (d, *J* = 8.1 Hz, 2H), 5.11 (d, *J* = 7.5 Hz, 1H), 3.24 (br s, 1H, OH).
*2,2-Dichloro-2-fluoro-1-(4-methoxyphenyl)ethanol* (**2e**). ^1^H-NMR (CDCl_3_): δ = 7.45 (d, *J* = 8.4 Hz, 2H), 6.92 (d, *J* = 8.4 Hz, 2H), 5.10 (d, *J* = 8.7 Hz, 1H), 3.82 (s, 3H), 2.97 (br s, 1H, OH).
*2*,*2-Dichloro-2-fluoro-1-(4-trifluoromethylphenyl)ethanol* (**2f**). ^1^H-NMR (CDCl_3_): δ = 7.51 (m, 4H), 5.3 (d, J = 8.1 Hz, 1H), 3.1 (br s, 1H, OH).
*2*,*2-Dichloro-2-fluoro-1-(4-fluorophenyl)ethanol* (**2g**). ^1^H-NMR (CDCl_3_): δ = 7.75 (m, 2H), 7.36 (m, 2H), 5.8 (d, *J* = 9.1 Hz, 1H), 3.3 (br s, 1H, OH).

## 4. Conclusions

In conclusion, we present a cost-effective and facile method for the addition and modification of aromatic aldehydes with CFCl_3_ under mild conditions. This method demonstrates a novel strategy for *in situ* formation of a reactive dichlorofluoromethyl magnesium Grignard reagent and its importance in synthesizing dichlorofluoromethyl aromatic alcohols. Unlike other previous methods, these reactions can be performed under relatively mild conditions and can also be used in reactions with de-activated or less electrophilic aromatic aldehyde substrates having electron donating substituents. However, the yields obtained under these conditions are moderate. This is attributed to the *in situ* formation of chlorofluorocarbene via a competing reaction pathway. Chlorofluorocarbene formation was verified via isolation of the cyclopropane product (**4**), 1,1,2,2-tetramethylchlorofluorocyclopropane formed from the reaction between tetramethylene (**3**) and chlrofluorocarbene. Based on previous reports [26,27,28,29], we conclude that chlorofluorocarbene formation could have occurred with equal probability under the employed reaction conditions, and further studies are needed to understand the kinetics and dependence of the various factors/parameters.
